# Normalization of Illumina Infinium whole-genome SNP data improves copy number estimates and allelic intensity ratios

**DOI:** 10.1186/1471-2105-9-409

**Published:** 2008-10-02

**Authors:** Johan Staaf, Johan Vallon-Christersson, David Lindgren, Gunnar Juliusson, Richard Rosenquist, Mattias Höglund, Åke Borg, Markus Ringnér

**Affiliations:** 1 Department of Oncology, Clinical Sciences, Lund University, SE-22185 Lund, Sweden; 2 CREATE Health Strategic Centre for Clinical Cancer Research, Lund University, SE-22184 Lund, Sweden; 3 Lund Strategic Research Center for Stem Cell Biology and Cell Therapy, Lund University, SE-22184 Lund, Sweden; 4 Department of Genetics and Pathology, Uppsala University, SE-75185 Uppsala, Sweden

## Abstract

**Background:**

Illumina Infinium whole genome genotyping (WGG) arrays are increasingly being applied in cancer genomics to study gene copy number alterations and allele-specific aberrations such as loss-of-heterozygosity (LOH). Methods developed for normalization of WGG arrays have mostly focused on diploid, normal samples. However, for cancer samples genomic aberrations may confound normalization and data interpretation. Therefore, we examined the effects of the conventionally used normalization method for Illumina Infinium arrays when applied to cancer samples.

**Results:**

We demonstrate an asymmetry in the detection of the two alleles for each SNP, which deleteriously influences both allelic proportions and copy number estimates. The asymmetry is caused by a remaining bias between the two dyes used in the Infinium II assay after using the normalization method in Illumina's proprietary software (BeadStudio). We propose a quantile normalization strategy for correction of this dye bias. We tested the normalization strategy using 535 individual hybridizations from 10 data sets from the analysis of cancer genomes and normal blood samples generated on Illumina Infinium II 300 k version 1 and 2, 370 k and 550 k BeadChips. We show that the proposed normalization strategy successfully removes asymmetry in estimates of both allelic proportions and copy numbers. Additionally, the normalization strategy reduces the technical variation for copy number estimates while retaining the response to copy number alterations.

**Conclusion:**

The proposed normalization strategy represents a valuable tool that improves the quality of data obtained from Illumina Infinium arrays, in particular when used for LOH and copy number variation studies.

## Background

Genomic copy number alterations (CNA) and allelic imbalances are common events in the development of cancer and certain genetic disorders [1,2]. The introduction of whole genome genotyping (WGG) arrays based on single nucleotide polymorphism (SNP) genotyping [3,4] allows for combined DNA copy number (SNP-CGH) and loss-of-heterozygosity (LOH) analysis at high resolution [5]. Currently, two major SNP array platforms are in use, Affymetrix GeneChip arrays [6] and Illumina BeadChips [7]. The Infinium assay for Illumina BeadChips is based on allele-specific hybridization coupled with primer extension of genomic DNA using primers directly surrounding the SNP on randomly ordered bead arrays [4]. The Infinium assay has been further developed into allele-specific single base extension using two color labeling with the Cy3 and Cy5 fluorescent dyes (Infinium II) [8]. Current generations of Infinium II arrays are able to interrogate more than 1 million SNPs simultaneously.

Infinium II is a two-channel assay and data consist of two intensity values (X, Y) for each SNP, with one intensity channel for each of the fluorescent dyes associated with the two alleles of the SNP. SNP markers are present at a high redundancy on Infinium II assays and the allele specific intensities (X, Y) are summarized estimates from replicate markers. The alleles measured by the X channel (Cy5 dye) are arbitrarily, with respect to haplotypes, called the A alleles, whereas the alleles measured by the Y channel (Cy3 dye) are called the B alleles. The allele specific intensities are normalized using a proprietary algorithm in the Illumina Beadstudio software. The normalization algorithm is applied on a sub-bead pool level and is designed to adjust for channel-dependent background and global intensity differences, and to scale the data. A sub-bead pool is a set of beads that were manufactured together and are located in roughly the same analytical location (stripe) on a BeadChip. The algorithm uses a 6-degree of freedom affine transformation with 5 main steps: outlier removal, background estimation, rotational estimation, shears estimation, and scaling estimation [5]. After normalization, data should be as canonical as possible with homozygous SNPs positioned along the transformed X and Y intensity axes. Normalized allele intensities are transformed to a combined SNP intensity, R (R = X + Y), and an allelic intensity ratio, theta (θ = 2/π*arctan(Y/X)).

R values are calibrated to generate copy number estimates (CN) by comparison to either a matched reference sample analyzed simultaneously or to canonical genotype clusters [5]. Canonical genotype clusters are generated from a large panel of normal samples and the clusters for a SNP indicate the R and theta values expected for each genotype (AA, AB and BB). Theta values are calibrated to generate B allele frequencies (BAF) using canonical genotype clusters. BAF is a value between 0 and 1 and represents the proportion contributed by one SNP allele (B) to the total copy number: BAF is an estimate of N_B_/(N_A_+ N_B_), where N_A _and N_B _are the number of A and B alleles, respectively. When canonical genotype clusters are used for calibration, copy number estimates are calculated per SNP by taking the log_2 _of the SNP intensity (R) divided by the SNP intensity expected from the canonical genotype clusters. Thus, copy number estimates may be regarded as a combination of two individual one-channel measurements of the amount of genetic material for a given SNP. Normalization of one-channel array data has been extensively explored, incorporating various algorithms, among which quantile normalization (QN) has been reported to perform consistently well [9] and has been widely used to normalize between arrays [10-12]. Recently, QN was applied, as one of several analysis steps, to Illumina Sentrix SNP BeadArrays to correct for an observed dye bias in copy number analysis [13].

Allelic imbalances in samples can be conveniently visualized in BAF plots [5]. A BAF value of 0.5 indicates a heterozygous genotype (AB), whereas 0 and 1 indicate homozygous genotypes (AA and BB, respectively). The allelic intensity ratio may, in the Infinium II assay, be regarded as a comparative dual channel measurement of the allelic proportion for a given SNP, similar to, e.g., two-channel gene expression data. Several reports have underlined the importance of intensity-based normalization, e.g., lowess [14], to correct for dye specific differences both for gene expression profiling [15,16] and array comparative genomic hybridization (aCGH) [17-19] in two-channel microarray data. Since alleles for SNPs are arbitrarily called A or B, a set of genomically consecutive SNPs will appear in BAF plots as horizontal bands that are expected to be symmetrically positioned around 0.5. For example, a region of single copy number gain in all cells will, in addition to the two bands of homozygous SNPs at BAF = 0 and BAF = 1, result in two bands: one at BAF = 0.33 with SNPs having genotype AAB and one at BAF = 0.67 with SNPs having genotype ABB.

Here we demonstrate that BAF plots for tumor samples analyzed on Infinium II BeadChips often display bands that are not symmetrically positioned around 0.5. We show that these asymmetrical allelic ratios are caused by a bias between the two dyes used in the Infinium II assay, and that this dye intensity bias also hampers copy number estimates. Dye-bias can potentially be both global and SNP-specific. We propose using a quantile normalization based strategy applied to summarized bead type data within arrays for global correction of this dye intensity bias. The strategy corrects asymmetries that remain between intensity channels after the conventionally used BeadStudio normalization for both allelic intensity ratios and copy number estimates in normal as well as in tumor samples. Note that whereas quantile normalization is widely applied to single channel arrays to normalize between arrays, we instead apply it to normalize between channels within Infinium II arrays. Of key importance for the success of the strategy is the generation of new normalized reference data sets for the calibration of theta and R into B allele frequency and log R ratio – the data set analyzed and the data set used for calibration should both be normalized in the same way. We investigated the performance of the normalization strategy using 535 individual hybridizations from 10 different data sets generated on four different Infinium II platforms. The investigated data sets contain normal blood samples as well as breast tumor, colon tumor, urothelial tumor and chronic lymphocytic leukemia (CLL) samples. The included tumors display a large number of different copy number imbalances, but also variation in tumor heterogeneity and normal cell contamination. We conclude that the normalization strategy improves Infinium II data for samples of many different types.

## Results and discussion

### Occurrence of asymmetrical B allele frequencies and copy number estimates in tumor specimens

**Figure 1:**
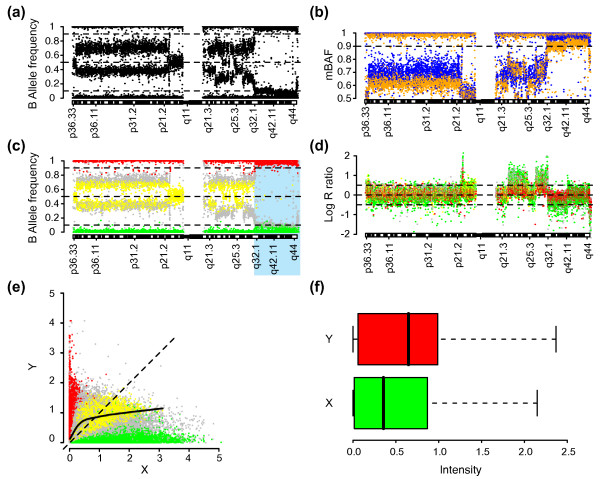
**Occurrence of asymmetrical B allele frequencies and copy number estimates.** Urothelial tumor UC152_I hybridized on an Infinium 370 k BeadChip is shown. CNV probes have been removed. (a) B allele frequency for chromosome 1. (b)Mirrored B allele frequency (mBAF) for chromosome 1, with individual SNPs colored according to BAF values: less than 0.5 (orange), above 0.5 (blue) showing the asymmetry of BAF values around 0.5. (c) BAF profile of chromosome 1, with individual SNPs colored according to genotype calls: AA (green), AB (yellow), BB (red) and no calls (gray). The cause of the BAF asymmetry also affects genotyping as seen for SNPs not assigned to a genotype (gray), which in the region 1q32.1 to qter (highlighted with a light blue background) predominantly are present with BAF < 0.5. (d) Copy number estimates (Log R ratio) for chromosome 1, with individual SNPs colored according to genotype. The cause of the BAF asymmetry also affects copy number estimates as seen for regions of gain and loss, where AA and BB SNPs do not overlap. (e)  Scatter plot of normalized allele intensities X and Y with individual SNPs colored according to genotype. A lowess regression line (solid) for heterozygous SNPs and the expected X = Y line (dashed) are superimposed. (f) Boxplots of the distributions of allele intensities X (green) and Y (red).

Allelic imbalances in tumor samples may conveniently be displayed using B allele frequency plots, which illustrate the presence and location of genomic regions of apparently the same allelic proportion (Figure 1a). In contrast to the expected symmetrical behavior of the B allele frequency, SNPs in regions of allelic imbalance appear to have bands of BAF values that are asymmetrically positioned around 0.5 for the analyzed urothelial tumor (Figure 1a). The asymmetry becomes even more apparent when a mirror transformation along the 0.5 axes of BAF to mBAF is performed (Figure 1b). Importantly, the asymmetry also affects genotyping, indicated by the higher number of failed genotype calls for lower BAF values (AA) compared to higher BAF values (BB) for the region 1q32.1 to qter (Figure 1c). In this region there are a total of 6421 SNPs evenly distributed between the upper BAF > 0.5 (3295 SNPs) and the lower BAF < 0.5 (3125 SNPs) parts. Of these 6421 SNPs, 927 SNPs have not been assigned a genotype by BeadStudio. 757 of these 927 failed calls have a BAF value below 0.5, showing that the cause of the observed asymmetry in BAF also affects genotyping. The BAF asymmetry also influences analysis methods for detecting allelic imbalance. Recently, the SOMATICs algorithm was proposed as a solution for detecting allelic imbalances in heterogeneous tumor samples using Infinium II data [20]. The algorithm divides the BAF profile into three bands (red, green, and blue) based on fixed BAF cut-offs. Asymmetry in BAF estimates causes regions of apparent identical allelic imbalance close to the fixed cut-offs to be identified in different bands (see Additional file 1). For copy number estimates asymmetry also exists for regions of CN loss and CN gain (Figure 1d). The asymmetry appears to be caused by an uncorrected curvature between the X and Y intensities for the two alleles (Figure 1e), and an unequal distribution of X and Y values (Figure 1f). We conclude that there seems to be a dye intensity bias between the two channels used in the Infinium II assay and that the bias remains after using the normalization in BeadStudio.

### Correction of dye intensity bias in HapMap samples using quantile normalization

Since the two alleles for each SNP are, with respect to haplotypes, arbitrarily associated with the X and Y intensities, normalized X and Y intensities should, in contrast to figure 1f, be expected to have essentially equal intensity distributions. Quantile normalization can be used to generate identical distributions from a set of distributions [9]. To investigate the effect of within sample QN of X and Y intensities from normal samples, we performed QN on X and Y intensities from HapMap samples used to generate the reference data sets for the Illumina 300 k version 1 (n = 111), 300 k version 2 (n = 120), 370 k (n = 123) and 550 k (n = 120) BeadChips. For each sample and SNP we calculated new normalized theta and R values thereby generating QN reference data sets. QN has been extensively used to normalize one-channel microarray expression data such that identical intensity distributions are generated for a set of arrays (between array normalization) [9]. Here we instead propose to use QN between channels within two-channel SNP arrays.

For each reference data set we computed new BAF and CN estimates and compared these estimates to BeadStudio data. Using QN we obtained CN estimates with significantly lower standard deviations (SD) for three of four reference data sets (Table 1). The mean decrease in SD for CN estimates was 15 – 26% for the 300 k v2, 370 k and 550 k data sets. For the Illumina 300 k v1 set, QN did not show any effect. Intriguingly, the single sample 300 k v1 BeadChips has a significantly lower variation of CN estimates than the Illumina version 2 Duo 300 k BeadChips (Table 1).

**Table 1:** Comparison of Log R ratio standard deviations between BeadStudio and quantile normalized HapMap data.

Platform	HapMap samples	Mean Log R ratio SD* BeadStudio	Mean Log R ratio SD* QN	p-value SD_QN _< SD_BeadStudio _**	Mean decrease in SD (%) ***
300 k v1	111	0.134	0.136	0.99	0
300 k v2	120	0.197	0.168	2.2*10^-16^	15
370 k	123	0.262	0.193	2.2*10^-16^	26
550 k	120	0.209	0.160	2.2*10^-16^	23

*: The SD of Log R ratio was calculated for each sample. The mean SD for all samples is shown.

**: Paired two-sided t-test. H0: Δ = mean Log R ratio SD_QN _– mean Log R ratio SD_BeadStudio _=0

***: (1- (mean Log R ratio SD_QN_)/(mean Log R ratio SD_BeadStudio_))*100

QN also showed a positive effect on allelic intensity ratios, generating lower standard deviations and more centralized theta positions for heterozygous SNPs (Table 2). Interestingly, it can be observed in table 2 that the average theta value for heterozygous SNPs differs from the expected 0.5 for all uncorrected and QN reference data sets. QN shows the least deviation from the expected value for all data sets, and also a clearly significant decrease in theta SD for samples across all data sets compared to BeadStudio data (Table 2).

**Table 2:** Comparison of effects on allelic intensity ratios between Illumina BeadStudio and quantile normalized HapMap data.

Platform	HapMap samples	Mean Theta_AB _± mean SD BeadStudio*	Mean Theta_AB _± mean SD QN*	Paired p-value theta SD_QN _< SD_BeadStduio _**
300 k v1	111	0.581 ± 0.095	0.454 ± 0.087	2.2*10^-16^
300 k v2	120	0.595 ± 0.097	0.457 ± 0.086	2.2*10^-16^
370 k	123	0.594 ± 0.097	0.460 ± 0.082	2.2*10^-16^
550 k	120	0.608 ± 0.099	0.451 ± 0.084	2.2*10^-16^

*: The mean and SD of theta was calculated for each sample. The mean of these values for all samples are shown.

**: Paired two-sided t-test. H0: Δ = SD_QN _- SD_BeadStudio _=0

### The intensity transformation introduced by QN can negatively affect allelic intensity ratio estimates

**Figure 2:**
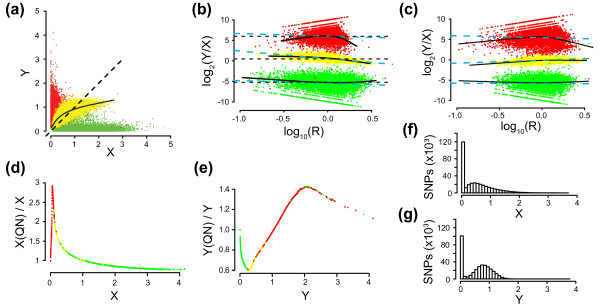
**Intensity transformations of X and Y by quantile normalization.** HapMap sample NA06985 hybridized on an Infinium 370 k BeadChip is shown. SNPs have been colored based on individual genotype calls: AA (green), AB (yellow), and BB (red). SNPs without genotype call are excluded. (a) Scatter plot of BeadStudio allele intensities X and Y. A lowess regression line for heterozygous SNPs is superimposed (solid) together with the expected X = Y line (dashed) illustrating that the dye intensity bias affects heterozygous SNPs. (b) MR plot of BeadStudio allele intensities for chromosome 8 with superimposed lowess regression lines (solid) for each genotype population and locally fitted linear regression lines (dashed blue). The mean M value for each genotype population is indicated by horizontally dashed black lines. (c) MR plot of quantile normalized allele intensities for chromosome 8 with superimposed lowess regression lines (solid black) and locally fitted linear regression lines (dashed blue) for each genotype population, separately. (d) Scatter plot of the intensity transformation X_QN_/X vs X from quantile normalization. SNPs are colored by genotype. SNPs with low X intensity values (predominantly genotyped as BB) are increased significantly in intensity by QN. (e) Scatter plot of the intensity transformation Y_QN_/Y vs Y from quantile normalization. SNPs are colored by genotype. (f) Histogram of BeadStudio X intensities. (g) Histogram of BeadStudioY intensities.

The deviation from theta = 0.5 for heterozygous SNPs in HapMap samples indicates that an imbalance in the X and Y intensity distributions remains after QN (Table 2). The imbalance in theta affects BAF estimates through the calibration of theta into BAF using the HapMap reference genotype clusters. Part of the imbalance can be explained by an uncorrected curvature between X and Y intensities that prior to QN is present for both tumor samples (Figure 1e) and HapMap samples (Figure 2a). To investigate the relationship between allelic intensity ratios and overall intensity we created MR plots where M = log_2_(Y/X) and R = log_10_(X + Y) similar to conventional MA plots [16]. Consequently, in MR plots heterozygote SNPs should have an M value of 0. As expected from figure 2a, curvature is present prior to QN in the MR plot of HapMap sample NA06985 for the AB, BB and AA SNP populations (Figure 2b). The curvature is highlighted by the superimposed lowess curve for each genotype population and the slope of a fitted linear regression line through each population. After QN there is less curvature, although not fully removed (Figure 2c).

To address how to improve QN, we investigated how QN transforms the X and Y intensities for HapMap sample NA06985 (Figure 2d and 2e). Low values of X are increased with relatively large factors in intensity, while Y values are generally decreased and scaled with smaller factors. SNPs with a low value of X are predominantly genotyped as BB, and the number of SNPs affected by the increase in X is large as seen by comparing the transformation (Figure 2d) with an X intensity histogram (Figure 2f). The same pattern is not observed for the Y intensity, for which the large majority of SNPs are transformed to a lower intensity (Figures 2e and 2g). Thus, QN introduces a transformation that results in a large increase for low X values, which affects a large number of SNPs.

**Figure 3:**
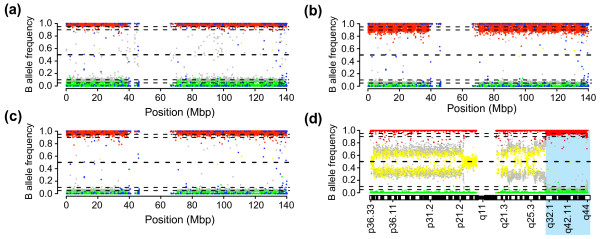
**Effects of quantile normalization on allelic intensity ratios.** Two urothelial carcinomas, UC456_R and UC152_I, analyzed using Infinium 370 k BeadChips are shown. SNPs have been colored based on individual genotype calls: AA (green), AB (yellow), BB (red), CNV probes (blue) and no calls (gray). Horizontal dashed lines represent BAF 0.05, 0.1, 0.5, 0.9 and 0.95, respectively. (a) BeadStudio normalized B allele frequency profile for chromosome 9 of UC456_R. (b) QN normalized B allele frequency profile for chromosome 9 of UC456_R. Compared to BeadStudio (a), QN increases variation for SNPs close to 1 in BAF and decreases variation for SNPs close to 0 in BAF. (c) tQN normalized B allele frequency profile for chromosome 9 of UC456_R. Application of a threshold for the increase in intensity of X and Y by QN lowers the variation of SNPs close to 1 in BAF compared to QN alone (b), and creates BAF values that are more symmetrical around BAF = 0.5 compared to BeadStudio (a). (d) tQN normalized B allele frequency profile for chromosome 1 of UC152_I. The region 1q32.1 to qter discussed in the text is highlighted with a light blue background. CNV probes have been removed.

The transformation imbalance does not appear to affect HapMap CN estimates for which the standard deviation is decreased in three of four reference data sets (Table 1). For CN estimates an increase of a low X value is not critical since the corresponding Y intensity is large and dominate the additive R value. However, an increase of low X values will cause more variation of the allelic ratios for SNPs with high values of Y (predominately genotyped as BB). An increase in the variation of allelic ratios for SNPs with low values of X will have the largest effect on regions with loss of allele A (thus dominated by Y with theta and BAF values close to 1). The impact of the transformation imbalance is further increased if the copy number loss is present in the absolute majority of investigated cells and not dampened by contaminating normal cells. To exemplify the effect of the transformation imbalance, the hemizygous loss of chromosome 9 in the urothelial carcinoma UC456_R is shown for both BeadStudio data (Figure 3a) and QN data (Figure 3b). While QN results in a reduced variation for SNPs with BAF values close to 0 (which have large X values), this improvement is counteracted with a large increase in the variation for SNPs with BAF values close to 1 (which have small X values). Furthermore, the transformation imbalance also appears to affect the correction of BAF asymmetry negatively.

### Incorporation of an intensity transformation threshold for QN improves allelic intensity ratio estimates

The negative effect of QN on allelic intensity ratios could potentially be circumvented by limiting the factor with which X intensity values are increased. Hence, we introduced a threshold for the QN intensity transformations to limit the increase of X and Y values before calculation of the allelic intensity ratio. In all our analyses, we used a threshold of 1.5 for the factor with which X and Y values could maximally be increased. While the threshold is applied identically to both X and Y transformations, it essentially only influences X values. A value of 1.5 appears reasonable as it incorporates the majority of SNPs with low X values (compare Figures 2d and 2e) without affecting the corresponding Y values, but the threshold may potentially be further improved by tuning. Using this QN modified with a threshold, tQN, we generated new tQN reference data sets. The application of the threshold markedly improved quantile normalized tumor BAF data by removing asymmetry and reducing variation (Figures 3c and 3d). Additionally, the removed asymmetry for allelic intensity ratios may provide a higher probability for SNPs to be genotyped, e.g., as AA for chromosome 9 of urothelial carcinoma UC456_R (Figure 3c compared to 3a) or as AA on 1q32.1 to qter for urothelial carcinoma UC152_I (Figure 3d compared to 1c). Consequently, tQN of Infinium II data could increase the genotype call rate, a variable commonly used to assess sample quality. An increased call rate for tumor specimens may also be beneficial for downstream LOH analysis software relying on genotype calls such as dChipSNP [21].

### Systematic investigation of BAF asymmetry in tumor samples before and after tQN

**Figure 4:**
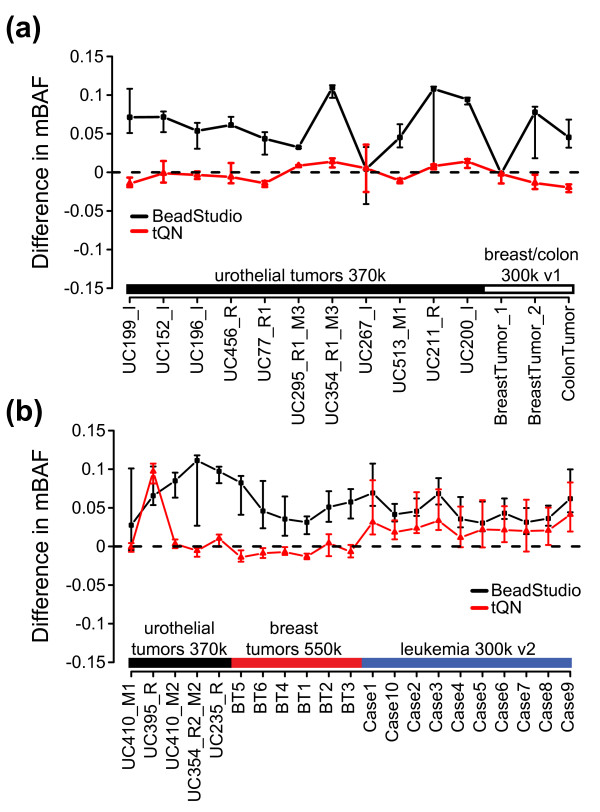
**Comparison of BAF asymmetry for regions of allelic imbalance before and after tQN across different Infinium II platforms.** BAF profiles for 35 tumor samples were divided into an upper (BAF > 0.5) and lower (BAF < 0.5) part, transformed to mBAF and separately segmented. For a defined genomic region, the average difference in segmented mBAF between the upper and lower part is expected to be zero if no asymmetry is present. Genomic regions were based on segmentation breakpoints of the upper BAF part. Only regions > 30 SNPs and with a segmented mBAF value > 0.6 in the upper and/or lower part were used in the comparisons. Black squares correspond to BeadStudio data and red triangles correspond to tQN data. Error bars for each sample and normalization method show the interquartile range (IQR). (a) BAF asymmetry for 14 matched tumor-normal samples. The black bar denotes 11 paired urothelial tumors from data set 4 and the white bar denotes the paired tumor samples from data set 8. tQN data systematically show less difference between the upper and lower BAF part compared to BeadStudio for the 14 matched tumors. (b) BAF asymmetry for 21 unmatched urothelial, breast and CLL tumor samples. The black bar denotes the 5 unpaired urothelial tumors from data set 4, the blue bar denotes CLL tumors from data set 7 and the red bar denotes breast tumors from data set 6. tQN data systematically show less difference between the upper and lower BAF part compared to BeadStudio for the 21 unmatched tumors.

To more comprehensively investigate BAF asymmetry before and after tQN, we divided 35 whole-genome tumor BAF profiles into an upper and lower part along the 0.5 axes. BAF values for each part were converted to mBAF, similar to figure 1b. Next, each part was separately segmented to find regions of consistent allelic proportion [22]. If no asymmetry is present for a defined genomic region the difference between segmented mBAF values for the upper and lower part of the BAF profile should be zero. We found that tQN results in significantly lower asymmetry for regions of apparent allelic imbalance in both paired and unpaired tumor samples across different Infinium II platforms (Figure 4). Essentially identical results were obtained irrespectively of which part of the BAF profile that was used to define the investigated regions. As expected from the upward shift of heterozygous theta positions (Table 2), the BeadStudio asymmetry is predominantly the result of higher mBAF values for the upper part of the BAF profile than for the lower part. This asymmetry is consistent with an upward shift of the entire BAF plot, as also observed in figures 1a and 3a. tQN showed the same positive effect on allelic intensity ratios for HapMap samples as shown for QN in table 2. For heterozygous SNPs, standard deviations were essentially identical for tQN and QN, whereas theta positions were marginally more centralized for tQN (data not shown).

### Effects of tQN on copy number estimates for tumor and normal samples

Having established that tQN corrects for asymmetry in allelic intensity ratio estimates, we investigated the effects of tQN on CN estimates compared to BeadStudio. To this aim, we applied tQN to Infinium II data sets containing both blood and tumor samples and performed three comparisons. First, we investigated whether tQN increase or decrease the response in log R ratio to CNAs. Second, we investigated if tQN decrease variation in CN estimates. Finally, we applied a CNV calling algorithm to tQN normalized HapMap data to investigate the overlap of identified regions compared to BeadStudio data.

**Figure 5:**
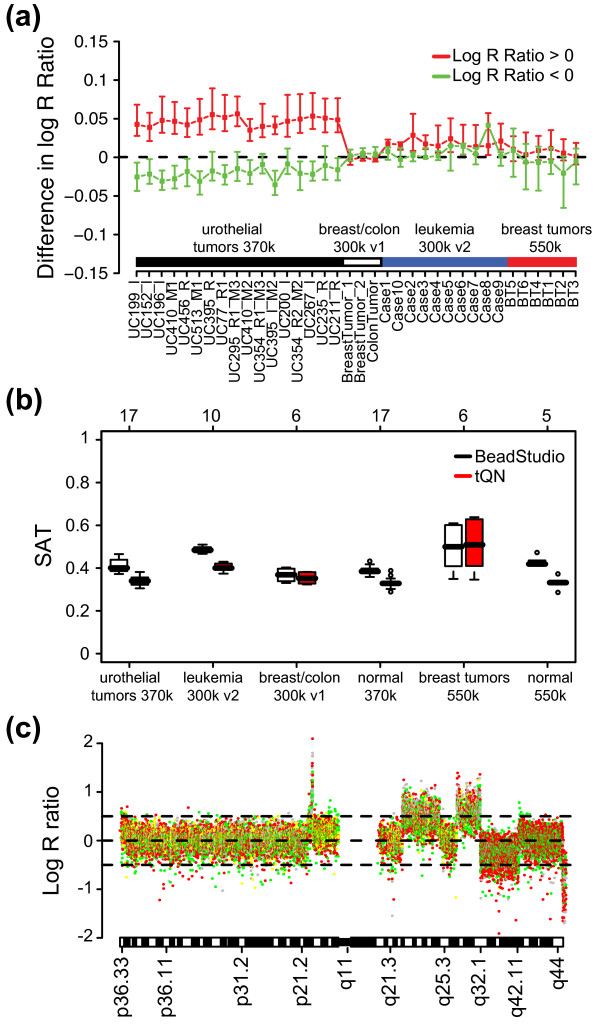
**Effects of tQN on copy number estimates across different Infinium platforms.** (a) Effect of tQN on log R ratio response to CNAs compared to BeadStudio data for 36 tumor samples. For each sample the mean difference in segmented log R ratio between BeadStudio and tQN data is plotted. For segments with log R ratio > 0 (red) the difference is BeadStudio minus tQN. For segments with log R ratio < 0 (green) the difference is tQN minus BeadStudio. A positive difference therefore corresponds to a better log R ratio response to CNAs for BeadStudio normalization compared to tQN for both types of segments. Error bars for each sample show the IQR of the difference. Horizontal bars denote the investigated data sets, urothelial tumors from data set 4 (black), breast/colon tumor samples from data set 8 (white), CLL samples from data set 7 (blue) and breast tumors from data set 6 (red). Only segments > 20 SNPs have been included. Segment definition was based on breakpoints from segmentation of BeadStudio copy number data. The small difference in segmented values between BeadStudio and tQN data indicates that tQN does not affect the log R ratio response to CNAs. (**b**) Boxplots of sample adaptive thresholds for BeadStudio normalized data (white) and tQN data (red) for 6 data sets. Top axis indicates the number of samples in each data set. tQN results in lower sample adaptive thresholds in four out of six data sets and equal thresholds in the remaining two. (**c**) tQN copy number estimates for chromosome 1 for urothelial tumor UC152_I with individual SNPs colored according to genotype calls: AA (green), AB (yellow), BB (red) and no calls (gray). CNV probes have been removed. tQN removes the asymmetry between AA and BB SNPs for regions of gain and loss observed in BeadStudio normalized data (compare to figure 1d).

To investigate whether tQN increase or decrease the response in log R ratios to CNAs compared to BeadStudio we applied segmentation to both tQN and BeadStudio tumor data. For each sample we calculated the difference in segmented log R ratios between BeadStudio data and tQN data. For genomic regions with log R ratio > 0 and < 0, respectively, the differences were calculated separately such that a positive difference for both types of regions corresponds to a better log R ratio response to CNAs for BeadStudio normalization compared to tQN. We observed small differences for all four data sets (Figure 5a). For the urothelial tumors, BeadStudio showed a better response for segments with gains, while tQN showed a better response for segments with losses. Such opposing findings indicate that the two methods result in different centering of the data rather than in different response to CNAs. Thus, tQN does not appear to alter the log R ratio response to CNAs compared to BeadStudio.

To investigate the effect on variation in CN estimates by tQN we computed sample adaptive noise thresholds (SATs) for tQN and BeadStudio data as previously described [18]. We obtained significantly lower SATs using tQN for four of six tested data sets, while SATs were essentially unchanged for the remaining two data sets (Figure 5b). The lack of effect by tQN on tumors hybridized on Infinium 300 k v1 BeadChips is in concordance with the reference data set (Table 1). The lack of improvement by tQN for tumors in the breast cancer data set is more difficult to explain. All tumors in this data set are either hyper-diploid or of high aneuploidy resulting in highly unbalanced CN profiles. Unbalanced CN profiles may be problematic for the affine transformation in BeadStudio, which scales the data based on that homozygous SNPs on average should exist in two copies, and therefore may confound normalization and data interpretation for aneuploid tumors [5]. A detailed investigation of this hypothesis is however not within the scope of the current study. The CLL data set is part of a comparison of four different array platforms for detection of CNAs and LOH [23]. In that study, Gunnarsson et al. compared the average copy number ratio and standard deviation for the normal chromosome 1 in all samples between the different platforms. The Illumina platform showed the highest average standard deviation (0.26) of the four platforms. We found that the average standard deviation for chromosome 1 after tQN was 0.21, which is comparable to the results obtained by Gunnarsson et al. for the Agilent platform (0.20). Furthermore, when applying tQN the asymmetry in CN estimates observed in figure 1d was removed (Figure 5c). The effect of tQN on CN and BAF estimates for various tumor and normal samples is further illustrated in Additional file 1. In conclusion, we find that tQN of Infinium II data is beneficial for CN estimates as variation is reduced while the dynamic response in copy number ratios to CNAs remains unchanged. A decreased variation for CN estimates can be beneficial for downstream analysis and detection of CNAs.

To investigate whether the reduced variation in copy number estimates by tQN affected CNV detection compared to BeadStudio we applied the PennCNV algorithm [24] to the HapMap 550 k reference data set. The overlap of identified SNPs between BeadStudio and tQN data was on average 80% across the 120 HapMap samples for CNV regions larger than 8 SNPs. Importantly, the overlap percentage increased for larger CNV regions. Even though we cannot validate the correctness of CNV regions identified in either tQN or BeadStudio data these findings indicate that tQN reduces noise without removing biologically relevant regions.

## Conclusion

We have developed a normalization method that improves the quality of data obtained from Illumina Infinium II genotyping arrays. We show that both allelic intensity ratio and copy number estimates are improved by using a quantile normalization strategy with a threshold for the intensity transformations (tQN) for correction of intensity dye bias when Infinium II BeadChips are applied to cancer samples. This dye bias results in an asymmetric detection of the two alleles for each SNP leading to asymmetry for both allelic intensity ratios and copy number estimates. Importantly, tQN not only removes such asymmetry but also reduces variation in copy number estimates. Essential for the improved result is to create reference data sets for calibration of B allele frequency and copy number estimates that are normalized with the same method that is applied to the investigated samples. The normalization strategy was successfully applied both to normal blood samples and tumor specimens with varying tumor heterogeneity and normal cell contamination. Our strategy is applied on a sample per sample basis and we have not evaluated if Infinium II data can be improved by using between array normalization. Further optimization of the normalization approach for Infinium II data should include adjusting X and Y intensities on a sub bead-level instead of the currently used summarized bead level to address the initially unequal X and Y distributions. Such a correction would presumably alleviate the need for an additional normalization. Potentially, such improvements may also address the lower ratio response to CNAs and signal to noise observed with SNP-CGH compared to conventional aCGH [23,25].

## Methods

### Data sets

We used 10 data sets for evaluation of the QN strategy. Data set 1 (HapMap 300 k v2) consists of 120 HapMap [26] samples hybridized on Illumina HumanHap300 version 2 Genotyping BeadChips (Courtesy of Illumina Inc., San Diego, CA). Data set 2 (HapMap 370 k) consists of 123 HapMap samples hybridized on Illumina HumanCNV370 Genotyping BeadChips (Courtesy of Illumina Inc.). Data set 3 (HapMap 550 k) consists of 120 HapMap samples hybridized on Illumina HumanHap550 Genotyping BeadChips (Courtesy of Illumina Inc.). Data set 4 (urothelial tumors 370 k) consists of 17 urothelial carcinomas hybridized on HumanCNV370 Genotyping BeadChips. Data set 5 (normal 370 k) consists of 17 normal samples hybridized on Illumina HumanCNV370 Genotyping BeadChips. Samples in data set 5 displayed call rates between 99.5 to 99.8%. Twelve of the samples in data sets 5 and 6 are paired tumor-normal samples from the same individual. Data set 6 (breast tumors 550 k) consists of six breast tumors hybridized on Illumina HumanHap550 Genotyping BeadChips. Data set 7 (leukemia 300 k v2) consists of ten CLL cases hybridized on Illumina HumanHap300 version 2 Genotyping BeadChips [23]. Data set 8 (breast/colon 300 k v1) consists of six hybridizations on Illumina HumanHap300 version 1 Genotyping BeadChips representing two breast cancers and one colon cancer with matching normal samples (Courtesy of Illumina Inc.). Data set 9 (HapMap 300 k v1) consists of 111 HapMap samples hybridized on Illumina HumanHap300 version 1 Genotyping BeadChips (Courtesy of Illumina Inc.). Data set 10 (normal 550 k) consists of one normal sample hybridized 5 times at different DNA concentrations on Illumina HumanHap550 Genotyping BeadChips (obtained from the PennCNV website [27]).

Chromosomes 1 to 22 were used in all comparisons. Data sets 4 and 5 were generated at the SCIBLU Genomics Centre at Lund University, Sweden [28] and data sets 6 and 7 at the SNP technology platform in Uppsala, Sweden [29] according to manufacturers instructions.

### BeadStudio data preprocessing

Fluorescent signals were imported into the BeadStudio software version 3.1 (Illumina Inc) and normalized. For each sample, the normalized fluorescence signal intensities were compared with the signal intensities of a set of reference genotypes, and the log_2_-ratios between sample and reference signals were calculated on a SNP per SNP basis. In addition, the frequency of the B-allele was for each sample estimated based on the reference genotype clusters [5]. Normalized X and Y intensities were exported for further analysis. Manifest used for 300 k version 2 BeadChips was HumanHap300v2_A. Manifest used for 300 k version 1 BeadChips was BDCHP-1x10-HUMANHAP300v1-1_11219278_C. Manifest used for 370 k BeadChips was HumanCNV370v1_C. Manifest used for 550 k BeadChips was HumanHap550v3_A. Mirrored B allele frequencies (mBAF) were calculated as mBAF = abs(BAF - 0.5) + 0.5 [22].

### Quantile normalization (tQN) of Infinium II data

tQN was performed individually for each sample using affine normalized intensities (X, Y) from BeadStudio and the R [30] package limma [31]. The combined SNP intensity, R, was calculated from tQN intensities. A threshold of 1.5 for the intensity transformations X_QN_/X and Y_QN_/Y was applied prior to calculation of theta: X_QN _intensities larger than 1.5 * X were set to 1.5 * X; Y_QN _intensities larger than 1.5 * Y were set to 1.5 * Y. Theta, B allele frequencies and copy number estimates were calculated from tQN normalized intensities and reference data sets as previously described [5]. CNV probes in analyzed samples were excluded from normalization due to lack of genotype information. Instead, for these probes the BeadStudio BAF and log R ratio values were used.

### Construction of tQN corrected reference data sets

Quantile normalized reference data sets were created from HapMap data sets using intensities (X, Y) normalized in BeadStudio as the starting point. For each sample and SNP, quantile normalized R and theta values were calculated as previously described [5]. Cluster positions in theta and R were calculated for each SNP and genotype based on genotype information (AA, AB and BB) using the mean of all samples for the specific SNP and genotype. SNPs with no cluster positions (no genotype assignment across all HapMap samples) were excluded from the analysis. BAF and copy number estimates for SNPs with only one genotype across all HapMap samples were calculated using the value of the single cluster position. Theta values for SNPs with one heterozygous and only one homozygous cluster position (e.g. AB and AA) were imputed for the missing homozygous cluster position (e.g. BB) by the median of all theta values for the missing genotype. Corresponding R estimates for the missing genotype were set as missing values. For CNV probes the original BeadStudio cluster positions were kept.

### Segmentation of allelic ratios for investigation of BAF asymmetry

For matched tumor-normal samples, SNPs homozygous in both the tumor and its matched normal sample were first removed from the tumor BAF profile. Next, each tumor sample was split into an upper and lower data set, based on BAF values > 0.5 or < 0.5. Both data sets were mirrored from BAF to mBAF (compare figure 1b) and segmented by CBS [32] using default settings as recently described [22]. Segments from the lower and upper part of the BAF profile were cross-mapped and the difference in the average segmented mBAF values between the upper and lower part for each genomic segment was calculated. If no asymmetry is present, the difference between the upper and lower part of the BAF profile should be zero. Only segments larger than 30 SNPs in size and with a segmented mBAF value > 0.6 in the upper and/or lower part was used for evaluation of asymmetry. For tumor samples without a matched normal, SNPs with BAF > 0.97 or < 0.03 were removed prior to splitting BAF profiles into an upper and lower part.

### Copy number analysis

Segmentation was performed on normalized Log R ratios for each sample, platform and method using CBS [32]. The significance level for accepting change-points, α, was set to 0.001 for all analyzed data sets and normalization methods. For comparisons between methods only segmented regions > 20 SNPs were used.

### Sample adaptive thresholds

Sample adaptive thresholds for CN estimates were calculated as previously described [18], using a smoothing window of 21 SNPs, the median of the SD distribution as cut-off, and a scaling factor of 2 for all analyzed data sets and normalization methods.

## Availability and requirements

Project name: tQN

Project home page: 

Operating system(s): Any operating system supporting Perl and R.

Programming language: Perl and R.

Other requirements: Perl modules File::Spec, Getopt::Long, IO::File and Pod::Usage. R package limma.

License: GNU GPL

Any restrictions to use by non-academics: None

Data set 6 (breast tumors 550 k) is available through NCBI's Gene Expression Omnibus [33] with accession GSE11977.

## Abbreviations

aCGH: array-based CGH; BAF: B allele frequency; CBS: circular binary segmentation; CGH: comparative genomic hybridization; CLL: chronic lymphocytic leukemia; CN: copy number; CNA: copy number aberration; CNV: copy number variation; IQR: interquartile range; LOH: loss of heterozygosity; mBAF: mirrored B allele frequency; QN: quantile normalization; SAT: sample adaptive threshold; SD: standard deviation; SNP: single nucleotide polymorphism; tQN: thresholded quantile normalization; WGG: whole genome genotyping.

## Authors' contributions

JS and MR conceived the study and developed the method. JS implemented the method and performed the analyses. JS and MR interpreted results and wrote the manuscript. JVC and DL contributed to discussions. GJ, RR, MH and ÅB contributed samples. All authors approved the final manuscript.

## Supplementary Material

Additional file 1 **Supplementary figures.** This file contains supplementary figures on the effect of BAF asymmetry on downstream analysis, a comparison of CN estimates before and after tQN, and a comparison of BAF asymmetry for regions of allelic imbalance before and after tQN. Click here for file

## References

[B1] Pinkel D, Albertson DG (2005). Comparative genomic hybridization. Annu Rev Genomics Hum Genet.

[B2] Rajagopalan H, Lengauer C (2004). Aneuploidy and cancer. Nature.

[B3] Matsuzaki H, Dong S, Loi H, Di X, Liu G, Hubbell E, Law J, Berntsen T, Chadha M, Hui H, Yang G, Kennedy GC, Webster TA, Cawley S, Walsh PS, Jones KW, Fodor SP, Mei R (2004). Genotyping over 100,000 SNPs on a pair of oligonucleotide arrays. Nat Methods.

[B4] Gunderson KL, Steemers FJ, Lee G, Mendoza LG, Chee MS (2005). A genome-wide scalable SNP genotyping assay using microarray technology. Nat Genet.

[B5] Peiffer DA, Le JM, Steemers FJ, Chang W, Jenniges T, Garcia F, Haden K, Li J, Shaw CA, Belmont J, Cheung SW, Shen RM, Barker DL, Gunderson KL (2006). High-resolution genomic profiling of chromosomal aberrations using Infinium whole-genome genotyping. Genome Res.

[B6] Affymetrix. http://www.affymetrix.com.

[B7] Illumina. http://www.illumina.com.

[B8] Steemers FJ, Chang W, Lee G, Barker DL, Shen R, Gunderson KL (2006). Whole-genome genotyping with the single-base extension assay. Nat Methods.

[B9] Bolstad BM, Irizarry RA, Astrand M, Speed TP (2003). A comparison of normalization methods for high density oligonucleotide array data based on variance and bias. Bioinformatics.

[B10] Barnes M, Freudenberg J, Thompson S, Aronow B, Pavlidis P (2005). Experimental comparison and cross-validation of the Affymetrix and Illumina gene expression analysis platforms. Nucleic Acids Res.

[B11] Carvalho B, Bengtsson H, Speed TP, Irizarry RA (2007). Exploration, normalization, and genotype calls of high-density oligonucleotide SNP array data. Biostatistics.

[B12] Dunning MJ, Barbosa-Morais NL, Lynch AG, Tavare S, Ritchie ME (2008). Statistical issues in the analysis of Illumina data. BMC Bioinformatics.

[B13] Oosting J, Lips EH, van Eijk R, Eilers PH, Szuhai K, Wijmenga C, Morreau H, van Wezel T (2007). High-resolution copy number analysis of paraffin-embedded archival tissue using SNP BeadArrays. Genome Res.

[B14] Yang YH, Dudoit S, Luu P, Lin DM, Peng V, Ngai J, Speed TP (2002). Normalization for cDNA microarray data: a robust composite method addressing single and multiple slide systematic variation. Nucleic Acids Res.

[B15] Quackenbush J (2002). Microarray data normalization and transformation. Nat Genet.

[B16] Smyth GK, Speed T (2003). Normalization of cDNA microarray data. Methods.

[B17] Khojasteh M, Lam WL, Ward RK, MacAulay C (2005). A stepwise framework for the normalization of array CGH data. BMC Bioinformatics.

[B18] Staaf J, Jonsson G, Ringner M, Vallon-Christersson J (2007). Normalization of array-CGH data: influence of copy number imbalances. BMC Genomics.

[B19] Neuvial P, Hupe P, Brito I, Liva S, Manie E, Brennetot C, Radvanyi F, Aurias A, Barillot E (2006). Spatial normalization of array-CGH data. BMC Bioinformatics.

[B20] Assie G, LaFramboise T, Platzer P, Bertherat J, Stratakis CA, Eng C (2008). SNP arrays in heterogeneous tissue: highly accurate collection of both germline and somatic genetic information from unpaired single tumor samples. Am J Hum Genet.

[B21] Lin M, Wei LJ, Sellers WR, Lieberfarb M, Wong WH, Li C (2004). dChipSNP: significance curve and clustering of SNP-array-based loss-of-heterozygosity data. Bioinformatics.

[B22] Staaf J, Lindgren D, Vallon-Christersson J, Isaksson A, Goransson H, Juliusson G, Rosenquist R, Hoglund M, Borg A, Ringner M (2008). Segmentation-based detection of allelic imbalance and loss-of-heterozygosity in cancer cells using whole genome SNP arrays. Genome Biol.

[B23] Gunnarsson R, Staaf J, Jansson M, Ottesen AM, Goransson H, Liljedahl U, Ralfkiaer U, Mansouri M, Buhl AM, Smedby KE, Hjalgrim H, Syvanen AC, Borg A, Isaksson A, Jurlander J, Juliusson G, Rosenquist R (2008). Screening for copy-number alterations and loss of heterozygosity in chronic lymphocytic leukemia-A comparative study of four differently designed, high resolution microarray platforms. Genes Chromosomes Cancer.

[B24] Wang K, Li M, Hadley D, Liu R, Glessner J, Grant SF, Hakonarson H, Bucan M (2007). PennCNV: an integrated hidden Markov model designed for high-resolution copy number variation detection in whole-genome SNP genotyping data. Genome Res.

[B25] Greshock J, Feng B, Nogueira C, Ivanova E, Perna I, Nathanson K, Protopopov A, Weber BL, Chin L (2007). A comparison of DNA copy number profiling platforms. Cancer Res.

[B26] HapMap. http://www.hapmap.org.

[B27] PennCNV. http://www.neurogenome.org/cnv/penncnv/.

[B28] SCIBLU Genomics, Lund University, Sweden. http://www.lth.se/sciblu.

[B29] SNP Technology Platform in Uppsala, Sweden. http://www.genotyping.se.

[B30] The R project for statistical computing. http://www.r-project.org.

[B31] BioConductor. http://www.bioconductor.org.

[B32] Venkatraman ES, Olshen AB (2007). A faster circular binary segmentation algorithm for the analysis of array CGH data. Bioinformatics.

[B33] Gene Expression Omnibus. http://www.ncbi.nlm.nih.gov/geo/.

