# Supplementary material for: Normalization of Illumina Infinium whole-genome SNP data improves copy number estimates and allelic intensity ratios
Source: BMC Bioinformatics. 2008 Oct 2;9:409. doi: 10.1186/1471-2105-9-409 (PMC2572624; doi:10.1186/1471-2105-9-409)

Additional file 1

Supplemental figure 1
Effect of BAF asymmetry in downstream analysis methods for detection of allelic imbalance. SOMATICs analysis of chromosome 9 of urothelial tumor UC199_I. No p-value filtering of identified segments has been applied. SNPs called as allelic imbalance by SOMATICs are colored based on SOMATICs band association (red, green, blue). Colors are overlaid in the order red, green and blue. The presence of asymmetrical B allele frequencies around BAF=0.5 introduces asymmetry in the SOMATICs band assignment, seen especially for the green band.

Supplemental figure 2
Comparison of CN estimates before and after tQN for urothelial tumors and normal samples hybridized on Infinium 370k BeadChips. SNPs are colored based on individual genotype calls: AA (green), AB (yellow), BB (red) and no calls (gray). In (a) and (b), the upper panel corresponds to BeadStudio estimates and the lower panel to tQN estimates. Genomic positions of SNPs are given in kb on x-axes. (a) CN estimates for part of chromosome 8 of urothelial tumor UC456_R. CNV probes are colored blue. tQN reduces the asymmetry in copy number estimates between AA and BB SNPs for amplified regions compared to BeadStudio. (b) CN estimates for chromosome 1 of normal sample UC288, for which tQN reduces the variation in copy number estimates compared to BeadStudio.

Supplemental figure 3
Comparison of BAF asymmetry for regions of allelic imbalance before and after tQN for breast cancer tumors hybridized on Infinium 550k BeadChips. SNPs are colored based on individual genotype calls: AA (green), AB (yellow), BB (red) and no calls (gray). In (a)-(c), the upper panel corresponds to BAF estimates from BeadStudio and the lower panel to BAF estimates from tQN. Horizontal dashed lines indicate BAF 0.03, 0.1, 0.5, 0.9 and 0.97, respectively. Genomic positions of SNPs are given in kb on x-axes. (a) BAF estimates for chromosome 6 of breast tumor BT6 before and after tQN. tQN reduces the B allele frequency asymmetry observed in BeadStudio normalized data for the different regions of allelic imbalance. (b) BAF estimates for chromosome 8 of breast tumor BT4 before and after tQN. tQN removes the gross asymmetry observed for SNPs genotyped as AB (yellow) in BeadStudio normalized data. (c) BAF estimates for chromosome 10 of breast tumor BT5 before and after tQN. tQN removes the B allele frequency asymmetry observed in BeadStudio normalized data for regions of allelic imbalance. Furthermore, SNPs genotyped as AB (yellow) are better centered at BAF = 0.5 for tQN data compared to BeadStudio.

Supplemental figure 1

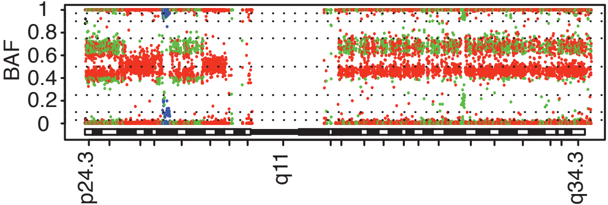

Supplemental figure 2

(a)

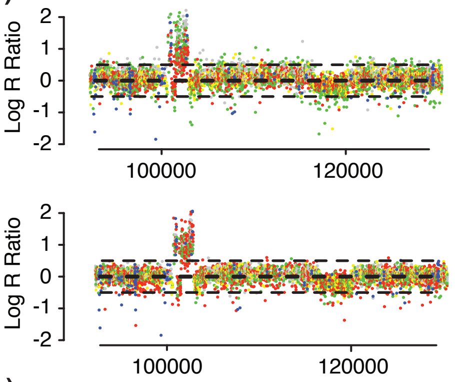

(b)

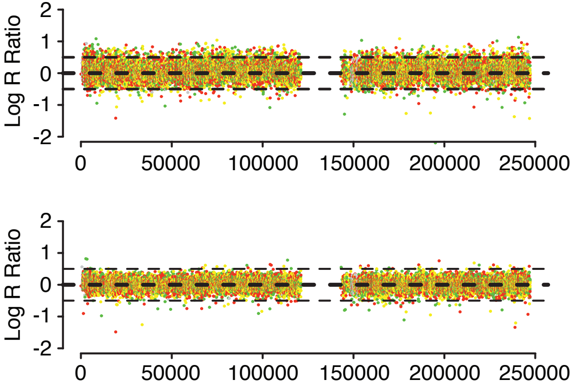

Supplemental figure 3

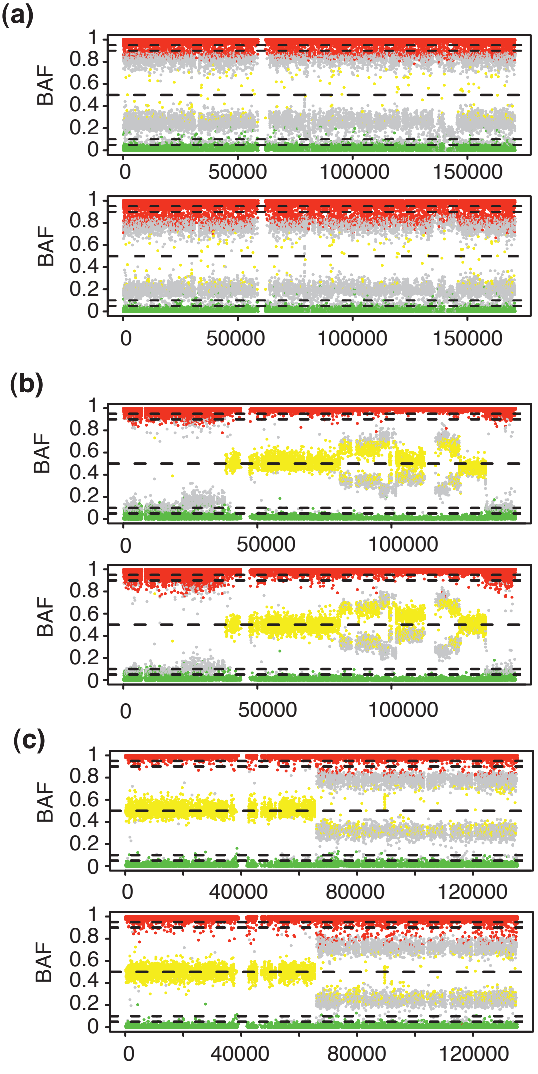

Supplement: Additional file 1 — Supplementary figures. This file contains supplementary figures on the effect of BAF asymmetry on downstream analysis, a comparison of CN estimates before and after tQN, and a comparison of BAF asymmetry for regions of allelic imbalance before and after tQN. [file 1471-2105-9-409-S1.pdf]
